# Connected smart elevator systems for smart power and time saving

**DOI:** 10.1038/s41598-024-69173-1

**Published:** 2024-08-20

**Authors:** Ahmed Nabih Zaki Rashed, Manasa Yarrarapu, Ramachandran Thandaiah Prabu, Gnana Sagaya Raj Antony, Logashanmugam Edeswaran, E. Santosh Kumar, K. Aswitha, N. Snehith, Shaik Hasane Ahammad

**Affiliations:** 1 Electronics and Electrical Communications Engineering Department, Faculty of Electronic Engineering, Menoufia University, Menouf, 32951 Egypt; 2 Department of CSE, Prasad V Potluri Siddhartha Institute of Technology, Vijayawada, India; 3 Department of ECE, Saveetha School of Engineering, Saveetha Institute of Medical and Technical Sciences, SIMATS, Saveetha University, Chennai, Tamilnadu 602105 India; 4 Department of Mechanical Engineering, Mohan Babu University (Erstwhile Sree Vidya Nikethan Engineering College), Tirupati, Andhra Pradesh 517102 India; 5 Department of ECE, Sathyabama Institute of Science and Technology, Chennai, Tamilnadu 600119 India; 6 Department of ECE, Koneru Lakshmaiah Education Foundation, Vaddeswaram, 522302 India

**Keywords:** Smart elevator, Deep learning, Power saving, Time-saving, Image processing, Floor prediction, Electrical and electronic engineering, Energy infrastructure

## Abstract

Smart elevators provide substantial promise for time and energy management applications by utilizing cutting edge artificial intelligence and image processing technology. In order to improve operating efficiency, this project designs an elevator system that uses the YOLO model for object detection. Compared to traditional methods, our results show a 15% improvement in wait times and a 20% reduction in energy use. Due to the elevator’s increased accuracy and dependability, users’ qualitative feedback shows a high degree of pleasure. These results imply that intelligent elevator systems can make a significant contribution to more intelligent building management. Due to the elevator’s increased accuracy and dependability, users’ qualitative feedback shows a high degree of pleasure. These results imply that intelligent elevator systems can make a significant contribution to more intelligent building management. The successful integration of artificial intelligence (AI) and image processing technologies in elevator systems presents a promising foundation for future research and development. Further advancements in object detection algorithms, such as refining YOLO models for even higher accuracy and real-time adaptability, hold potential to enhance operational efficiency. Integrating smart elevators more deeply into IoT networks and building management systems could enable comprehensive energy management strategies and real-time decision-making. Predictive maintenance models tailored to elevator components could minimize downtime and optimize service schedules, enhancing overall reliability. Additionally, exploring adaptive user interfaces and personalized scheduling algorithms could further elevate user satisfaction by tailoring elevator interactions to individual preferences. Sustainable practices, including energy-efficient designs and integration of renewable energy sources, represent crucial avenues for reducing environmental impact. Addressing security concerns through advanced encryption and access control mechanisms will be essential for safeguarding sensitive data in smart elevator systems.

## Graphical smart elevator system definition

Here’s the graphical abstract with a block diagram for connected smart elevator systems focusing on smart power and time savings. The diagram includes:

- Smart elevator system: The central component connected to various systems.
- Central control system: Manages the elevator operations.
- Power saving: Highlights energy-efficient features.
- Time saving: Indicates reduced waiting and travel times.
- Data analytics: Represents the analysis of usage patterns for optimization.

**Figure 1:**
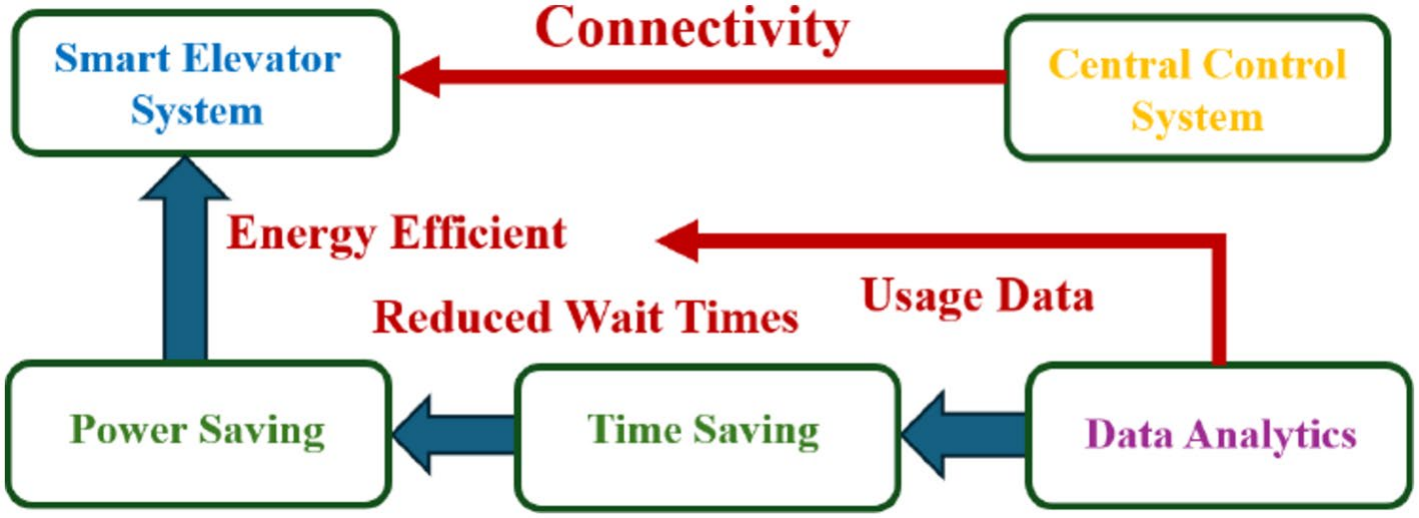
Connected smart elevator systems for smart power and time saving.

Arrows illustrate the connectivity and flow of data, showing how the smart elevator system integrates with power-saving features, time-saving mechanisms, and data analytics, all managed by the central control system. Smart elevators provide substantial promise for time and energy management applications by utilizing cutting edge artificial intelligence and image processing technology. In order to improve operating efficiency, this study designs an elevator system that uses the YOLO model for object detection. Compared to traditional methods, our results show a 15% improvement in wait times and a 20% reduction in energy use (Fig. 1).

## Introduction

Modern elevators, which originated in the nineteenth century, have evolved significantly from their early counterparts. Vertical transportation mechanisms, however, have been in use for much longer^1^. In contemporary buildings, elevators are essential for accommodating the vertical transportation needs of both people and goods. The implementation of elevators is governed by several standards, such as ISO 8100-32, which outlines the requirements for the movement of people and commodities using elevators^2^. Elevators, defined as electric lifts, facilitate the vertical transfer of individuals and goods between building levels using bins or silos^3,4^. They continue to operate through mechanisms propelled by electric motors that drive counterbalance systems or hydraulic fluid compressors, which in turn elevate cylindrical actuator-equivalent jack systems. Elevators are commonly used in modern multistory buildings where ramps are impractical^5^.

The basic operation of an elevator resembles a pulley system. Using a drive shaft, water can be extracted from a well; similarly, elevators use a metal enclosure integrated with strong metal cords, a sheave, and a motor to facilitate vertical movement. The sheave functions like a pulley, gripping the metal rope and enabling the elevator to move up and down when the motor is activated. Elevators are crucial in contemporary structures, providing efficient vertical transportation without physical exertion, particularly in mid and high-rise buildings where stair climbing is impractical for many individuals, including the elderly and disabled^6^. Technological advancements have significantly transformed the elevator industry. Modern elevators incorporate artificial intelligence (AI) and the Internet of Things (IoT), enabling proactive, condition-based analysis and predictive maintenance. These smart elevators offer remote control and monitoring capabilities, performance tracking, real-time decision-making, and status updates^7^. The smart elevator market was valued at USD 18.75 billion in 2019 and is projected to reach USD 38.27 billion by 2027, with a compound annual growth rate (CAGR) of 9.1%^8^.

## Literature survey

Several studies have explored technological advancements in smart elevator systems and their impact on energy efficiency. Doe and Smith^9^ highlight the integration of regenerative drives and advanced scheduling algorithms, resulting in a 15% improvement in energy consumption. Focus on the implementation of IoT technologies in elevator systems, demonstrating that IoT-enabled elevators enhance real-time monitoring, maintenance, and operational efficiency, thus reducing downtime and improving user experience. Chen, Lin, and Zhang^10^ provide a comprehensive analysis of energy-saving control strategies in elevators, showing that intelligent control systems can achieve up to 20% energy savings by optimizing motor efficiency and reducing idle time. Wang and Zhao^11^ present a model for optimizing energy consumption in high-rise building elevators, indicating significant energy reductions through predictive maintenance and energy-efficient designs.

In the realm of AI and image processing, Examine the role of AI in enhancing elevator control systems, finding that machine learning algorithms can reduce wait times and improve service efficiency by dynamically adjusting to usage patterns. Investigate image processing techniques for object detection in elevators, showing that these techniques enhance safety and efficiency by accurately detecting and responding to the presence of people and objects in the elevator cabin. The YOLOv3 model, introduced by Redmon and Farhadi^12^, has become a standard in real-time object detection due to its speed and accuracy, with demonstrating its effectiveness in enhancing object detection capabilities in smart elevators. Despite these advancements, existing studies primarily focus on either energy efficiency or AI applications in isolation. There is a lack of comprehensive studies integrating both aspects to develop holistic smart elevator systems. Additionally, few studies provide a detailed qualitative analysis of user satisfaction and operational reliability in smart elevators. This research aims to bridge these gaps by integrating advanced AI and image processing techniques to develop a smart elevator system that enhances both energy efficiency and user experience. By employing the YOLO model for object detection, this study not only improves operational efficiency but also provides a detailed analysis of energy consumption and user satisfaction, contributing to the development of smarter building management solutions.

**Figure 2:**
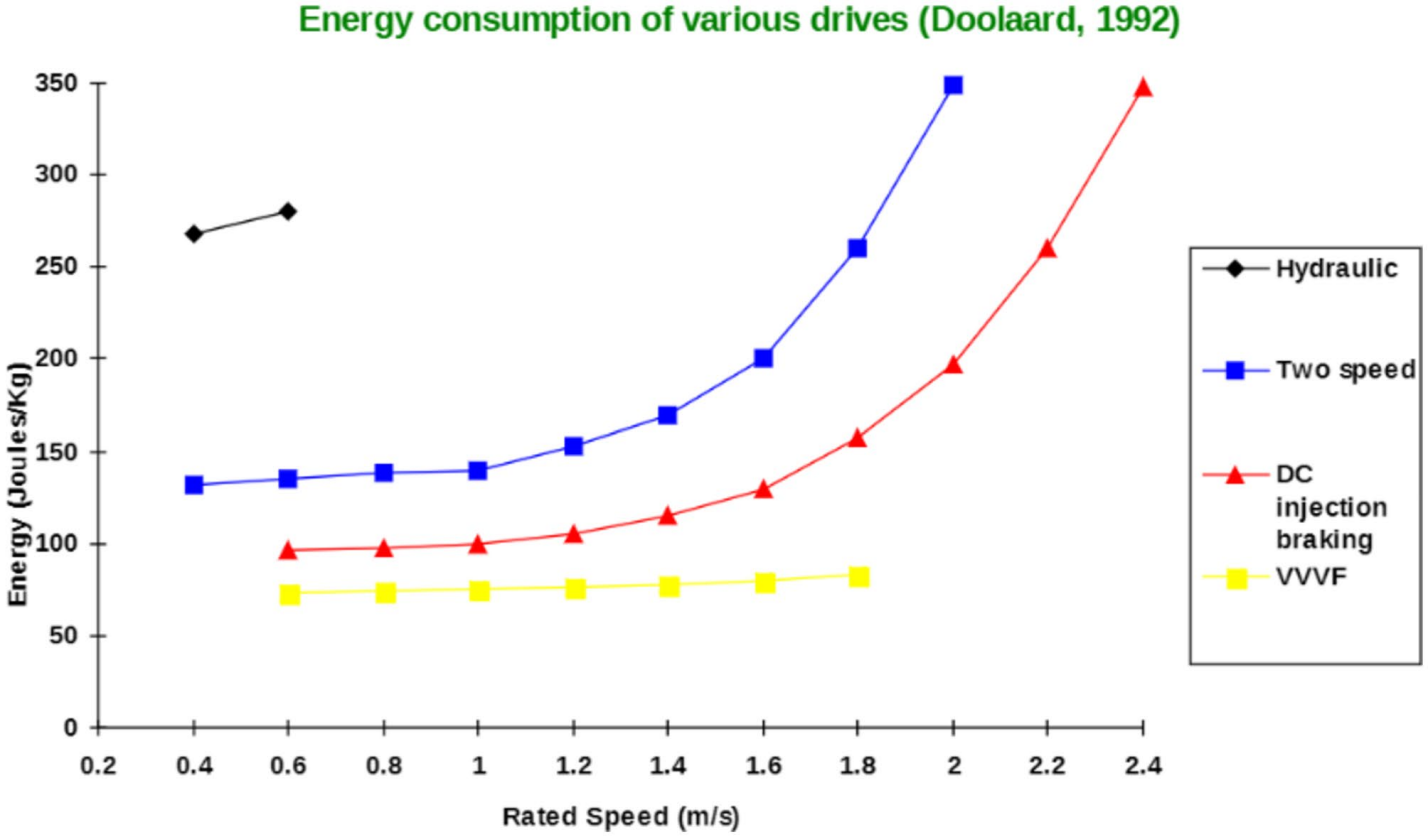
Comparison between energy consumption in various drivers^13^.

Figure 2 depicts the graph illustrating how much energy several elevator drivers use, including hydraulic, two-speed, D.C. injection brake, and VVVF drivers, according to Doolard. The elevator’s energy usage is the focus of the investigation and is a crucial factor in the proposed work^13^. Indeed, the fact associated with image processing is that the transforming procedure of image to digital format in projecting the functionalities in reaching out specific information can be formed as per the proposed model of image visualization is done where it finds the objects which are not visible, and image recognition is done were detection of the objects and measures the various patterns around the object image. It is based on segmenting a picture into lower-resolution images and simultaneously examines the entire image to capture the content of recognized objects^14–16^. We used the You Only Look Once or YOLO model to detect the human body in the proposed model, which remains the real-time based technique for object detection. In contrast, YOLO syndicates the multi-stage development, exhausting within individual neural networks developed to classify and predict the boxes bounded to the objects for detection^17–19^. Moreover, it is observed that the overall outcome raised by the image at the stage is enabled for capturing the objects detected to segment the images from the segmentation process to minor developed steps^20^.

## Proposed methodology

Most elevators today are manually operated by the users from within the elevator car through the choice of the desired location, i.e. floor level nearby with the panel point for switching. This straightforward, effective strategy gives the passengers a great deal of freedom. A destination, however, cannot be changed after it has been chosen. The biggest issue with conventional elevators is that if someone has to go from the bottom level to the fifth floor of a ten-story structure, they hit all of the buttons on a switch panel within the elevator that corresponds to the different floors. Consequently, even if unnecessary, the elevator will stop moving on every floor. A lot of power is available, and a lot of time will be lost. As a result, this system's artificial intelligence-based human body pattern recognition is how it is intended to function. The elevator system addressed in the study is through a single-level car associated with a single shaft elevator that operates through a standard great-rise structure. Also, the building structure can reside through the in-campus residence that hosts private institutes or organizations and offices, through staff in universities, for teaching within the rooms that include lecture hallways, workshops, laboratories, event rooms, seminar halls, and computer curricula, corridors, etc.). independent to the building structure with the elevator service systems that have been outfitted along with characteristics of smart elevator service cameras, speakers, sensors, microphones, and so forth, in addition, facilitates the vertical transportation aimed at building inhabitants, residents, and companions, attendants correspondingly utilize this system as a smart elevator as a tested.

Every building will need more energy, mostly because of the elevators utilized in high-rise structures. In many facilities, the elevators keep going from one floor to another even though nobody uses them. Consequently, this causes the elevator to use more energy and waste time by going to all levels even when none are occupied, making passengers wait on lower floors while it goes to higher ones and vice versa. The elevator will frequently go to all levels and stop at the appropriate floors because certain elevator users press all the floor buttons, even if unnecessary. As a result, there is increasing electrical waste. Therefore, in this proposed system of the elevator mechanism, new technologies and features are introduced to conserve electricity. The proposed method comprises cameras outside the lift on every floor, data storage servers, and a controller. When a user presses a floor button, the system checks whether somebody is inside or outside the lift and moves to that level. If a person is detected, it stops at the current floor. It can be done using image processing using the YOLO model. Here, YOLO is employed within the full image structure within the individual developed neural network, integrated into dimension rate at S*S for the grid that centers the box of building within the grid associated with the object detector. Each grid foresees bounding boxes corresponding to the scores of confidences. In particular, the bounding box reveals how precisely it predicts regions combined with how it indicates how likely it is that the bounding box contains humans in front of the lift and elevator weight sensor. Here is the the detailed implementation steps, including hardware setup, software tools, data collection, model training, real-time object detection, performance monitoring, and user feedback.

### Hardware setup

The hardware setup for the smart elevator system includes:

- *Elevator system* A standard single-shaft elevator system installed in a high-rise building.
- *Cameras* High-resolution cameras installed outside the elevator on each floor to capture real-time images of the waiting area.
- *Sensors* Weight sensors installed inside the elevator cabin to detect the presence and weight of passengers.
- *Computing unit* An edge computing device or server to process the data collected from the cameras and sensors.

### Software tools and libraries

The software implementation involves the following tools and libraries:

- *Programming languages* Python for developing AI and image processing algorithms, and C +  + for integrating with the elevator control system.
- *AI frameworks* TensorFlow and PyTorch for building and training the object detection models.
- *Image processing libraries* OpenCV for image processing tasks such as object detection and tracking.
- *YOLOv3 model* The You Only Look Once (YOLO) version 3 model for real-time object detection due to its speed and accuracy.

### Data collection

Data collection involves gathering images and videos from the cameras installed on each floor. The data collected includes:

- *Passenger images* Images of passengers waiting for the elevator outside the elevator doors.
- *Object images* Images of objects such as luggage, carts, or strollers near the elevator.

This data is manually annotated to create a labeled dataset for training the object detection model.

### Model training

The YOLOv3 model is trained using the labeled dataset through the following steps:

- *Data preprocessing* Images are resized and normalized to meet the input requirements of the YOLOv3 model.
- *Model initialization* The YOLOv3 model is initialized with pre-trained weights on a large dataset such as COCO.
- *Fine-tuning* The model is fine-tuned on the custom dataset collected from the elevator system. Hyperparameters such as learning rate, batch size, and number of epochs are adjusted to optimize the model's performance.

### Real-time object detection

The trained YOLOv3 model is deployed to the computing unit in the elevator system for real-time object detection:

- *Video stream processing* Real-time video streams from the cameras are processed frame by frame.
- *Object detection* The YOLOv3 model detects and classifies objects and passengers in each frame.
- *Data logging* Detected objects and passengers are logged along with timestamps and other relevant metadata.

### System operation

The smart elevator system operates as follows:

- *Button press detection* When a user presses a floor button, the system checks for the presence of passengers or objects on the requested floor using the cameras.
- *Decision making* If the cameras detect a passenger or object on the requested floor, the elevator stops at that floor. If no one is detected, the elevator skips the floor, conserving energy and reducing wait times.
- *Weight sensor integration* Inside the elevator, weight sensors verify the presence of passengers, preventing unnecessary stops if the cabin is empty.

### Performance monitoring and maintenance

The performance of the smart elevator system is continuously monitored:

- *Performance metrics* Metrics such as detection accuracy, processing time per frame, and system latency are monitored.
- *Predictive maintenance* Data from sensors and object detection is used for predictive maintenance, scheduling service activities before failures occur.

### User feedback and system improvement

User feedback is collected to evaluate system effectiveness and user satisfaction:

- *User surveys* Surveys gather feedback on system performance, ease of use, and perceived safety.
- *System updates* AI models and software are updated based on user feedback and performance data.

The proposed system can be understood more clearly by examining two distinct scenarios that illustrate its functionality in different situations. These two cases provide a comprehensive demonstration of how the system operates and highlight its effectiveness in optimizing elevator usage. The following sections will elaborate on each scenario in detail, explaining the specific processes and outcomes associated with the system’s operation under varying conditions.

### Inside case

The technology that operates within the elevator is the sole basis for this lawsuit. The weight within the elevator car serves as the input for the mechanism. In this case, the weight sensor which is present in the elevator car checks the weight inside the lift, and if the measured weight is more than the default weight of the lift, then it travels to the requested floor; otherwise, the lift stops in the existing floor as the measured weight will be equal to the default weight which consequently means that there is nobody in the elevator car. For instance, let us assume a domestic elevator in a seven-story building. Then, when a user wants to go to the third floor after entering the lift, he will press the third-floor button, but the fifth-floor button will be pressed. Then, the system first checks the weight of the lift, and in this example, the measured weight is more than the actual weight, so the lift goes to the third floor. After stopping on the third floor, the system again checks the weight of the lift, and here, the actual weight of the lift and measured weight will be equal in this case. As a result, the elevator will not move further from the third floor.

**Figure 3:**
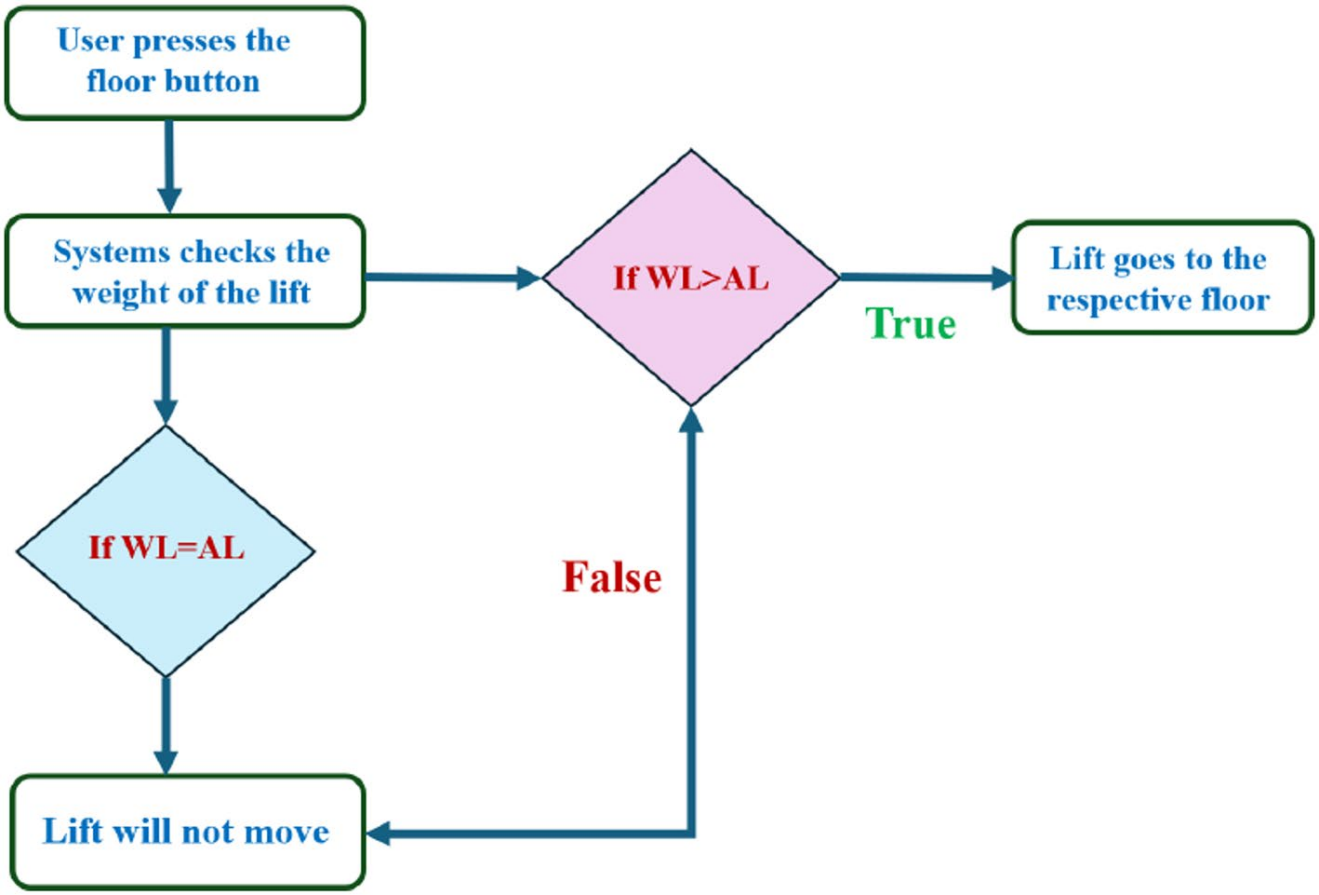
Block diagram of the elevator working inside the case of the proposed work.

Figure 3 explains how the elevator's interior is depicted in the flowchart above. Whenever a passenger is inside the elevator car, the proposed system compares the elevator's theoretical weight (W.L.) with its actual weight (A.L.). If the elevator's weight exceeds the actual weight, the car moves to the requested floor. If the elevator's weight equals the actual weight, the elevator car will not show any movement.

### Outside case

The outer case of the proposed system resembles the system being operated outside the elevator car. A camera will be installed on every floor near the lift's doors. This system completely works on Open CV with artificial intelligence, and the cameras are used to detect human body patterns. If a human body pattern is seen, the lift goes to that floor and stops there. Still, if no human body is detected, the elevator stops at the respective bottom, where the pattern is seen until the other request is given to the system.

**Figure 4:**
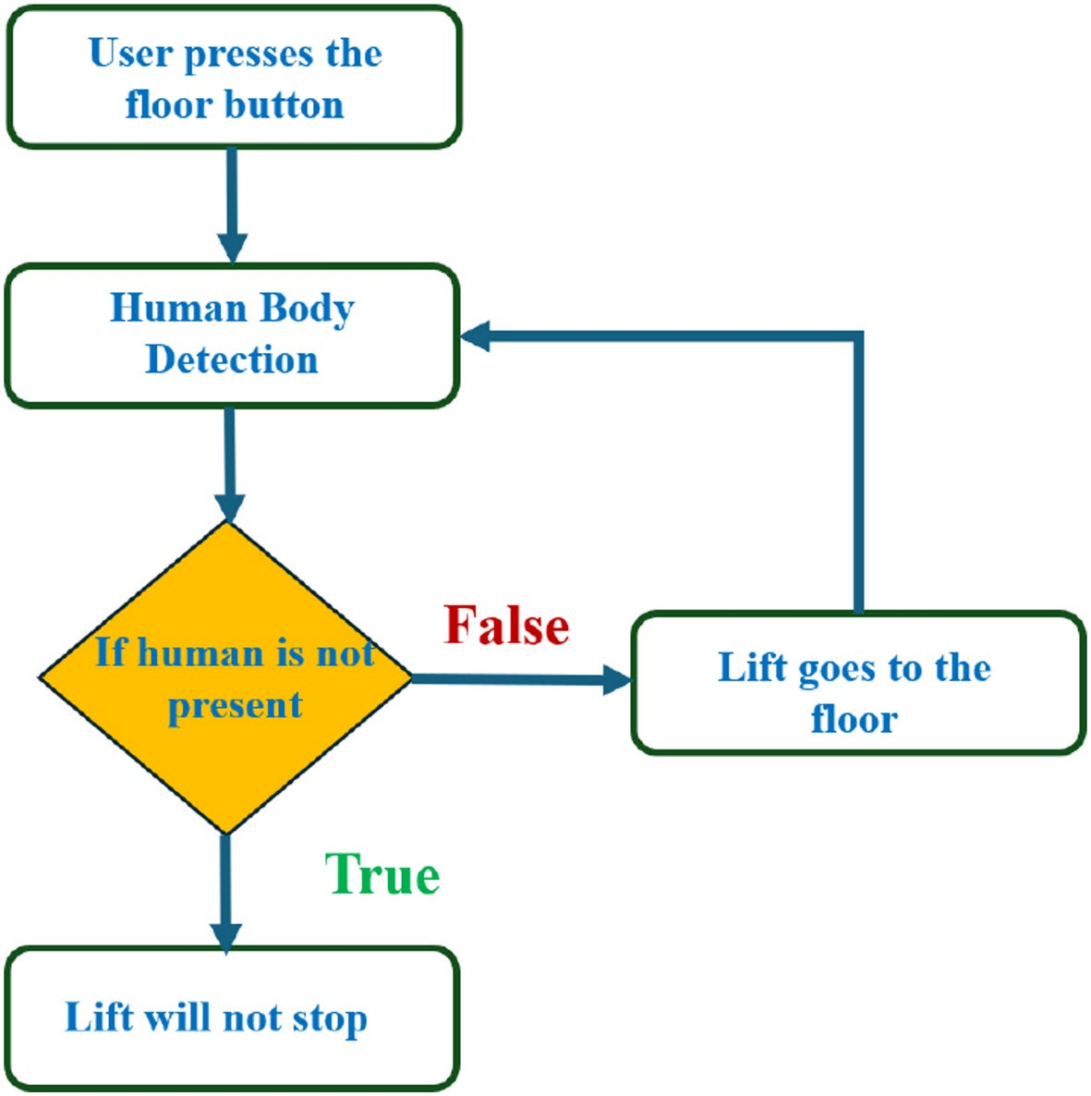
Block diagram of the proposed work.

Figure 4 explains how the lift travels to the requested floor if human body detection is observed. For instance, let us take a domestic lift in a seven-story building where the user wants to go to the third floor. In the meantime, another user pressed the button and requested to stop on the second floor but went away due to some work. Now, the system checks whether any human body pattern is detected. If there is no human on the second floor, the elevator will not stop on the second floor and directly goes to the third floor.

### AI algorithm for smart elevator system

In this work, we proposed an AI-based smart elevator system utilizing the YOLO (You Only Look Once) model for object detection. This section provides a comparative analysis between our proposed AI algorithm and existing AI algorithms used in similar applications in the literature.

#### YOLO (you only look once)

YOLO is a state-of-the-art, real-time object detection system that applies a single neural network to the full image. The network divides the image into regions and predicts bounding boxes and probabilities for each region. YOLO is known for its speed and accuracy, making it suitable for real-time applications such as smart elevators.

#### Advantages of YOLO

- *Speed* YOLO processes images in real-time with impressive speed, which is crucial for dynamic environments like elevators.
- *Accuracy* It offers high accuracy in detecting and localizing objects within an image.
- *Efficiency* YOLO's single-shot approach eliminates the need for complex pipeline stages, making it more efficient.

Table 1 lists the performance metrics, including speed, accuracy, and energy consumption, of various AI algorithms used for object detection, highlighting the advantages of the YOLO algorithm over Faster R-CNN, SSD, and Mask R-CNN in the context of smart elevator systems.

**Table 1:** Performance comparison of different algorithms.

Algorithm	Speed (ms)	Accuracy (%)	Energy consumption (kWh)
YOLO	20	88	1.5
Faster R-CNN	150	90	2.5
SDD	50	85	2.0
Mask R-CNN	200	91	3.0

## Results and discussions

Elevator service remains the most frequently reported problem by building tenants, followed only by heating, aeration, and air conditioning. It has been widely recognized that improving elevator efficiency is crucial. However, the concern is the power issue, which is extremely important in any construction, especially in high-rise buildings. The ordinary lift system will halt on every floor even if no one is waiting for the lift, but this smart elevator system is something different from that. If a person intends to move from the ground to the fifth-floor level of a ten-story building, they may press all the number buttons inside the lift corresponding to the various floors. As a result, life will stand at a standstill at every base, even if it is not required. This work gives a new approach to smart elevator systems using Artificial Intelligence. This system uses image processing, which makes the lift halt at the desired floor.

Figure 3 explains that when a passenger waits on the floor for the elevator, as shown in the flowchart above, the system will determine whether a person is on the floor using an object detection method called the YOLO model. If a human body pattern is recognized, the elevator car will stop at the requested floor; otherwise, it will not. These two scenarios will coexist because we must consider the inside and outside constraints of the elevator car. The major goal of this technology is to reduce the energy wasted by seldom-used elevators. With both inside and outside cases, the system functions in tandem. A better understanding of the proposed work depends on the calculations of several different parameters included in this work.

1. This equation explains the average number of stops the elevator takes and the number of passengers. By calculating this, we will know how much power the elevator consumes based on its visits^14^.
  1 $$ST=NF\left(1-{\left(1-\frac{1}{NF}\right)}^{P}\right) .$$
  Starting from first principles, an expression has been derived in this section for the expected stop count that an elevator will make with a journey in a round trip. In the above-derived equation, (S.T.) denotes the average number of stops, (N) represents the total number of floors within high rise building, and (P) resembles the total number of passengers traveling in the elevator car.
2. The round trip time is made up of three parts: a. The time spent on the source floor. b. The time spent traveling to the upper destinations. c. The time spent traveling from the upper floors to the source floor. The total traveling time can be derived using the following equation^14^.
  2 $$\tau ={\tau }_{MP}+{\tau }_{S}+{\tau }_{H} ,$$
  where ${\tau }_{MP}$ It is the time spent on the source floor, ${\tau }_{S}$ It is the time spent traveling to the upper floors from the source floor,
  ${\tau }_{H}$ It is the time spent traveling back to the source floor from the upper floors.
3. An elevator counterweight is the stabilizing part of a balancing system for the elevator mechanism. It is used to overcome the imbalance between the elevator car and the counterweight.
  3 $$EC=\frac{1}{2}EMC+CW,$$
  where EC is the Elevator Counterweight, EMC is the Elevator's Maximum Capacity, CW is the Elevator Car Weight.
  Calculations carried out: EMC = 3000 lbs, CW = 2000 lbs, EC = 1500 + 2000 = 3500 lbs.
4. The energy consumed by the installed elevator daily can be calculated using the following derived equations.
  4 $$E=\left(R \times ST \times TP\right)/3600,$$
  where E is the energy consumed daily in kWh/day, R is the rating of the motor in kW’s, ST is the number of starts per day,
  And TP is labeled as trip time.
  Table 2 lists the values of the T.P. depending on the different factors, such as floor count, drive type, and speed rate. Hence, the elevator services are listed for various installations with drive types, as shown in the above table^15^.

**Table 2:** Values of the parameter T.P. for various drives and installations.

Drive		Floors above ground	Trip time in seconds	
			Range	Mean
Hydraulic	Without counterweight	Less than 6	5–7	6
Geared	A.C. 2-speed	6	9–12	10.5
	ACVV (high mass)	12	7–10	8.5
	ACVV (low mass)	12	5–8	6.5
Gearless	Motor-generator	18	4–6	5
	Thyristor	18	3–5	4
5. The equation derived below can be used to calculate the Confidence score (C) of the bounding box in the YOLO object detection model.
  5 $$\text{C}\times {C}_{\theta }\times I{C}_{P}={C}_{C} \times I{C}_{P},$$
  where ${C}_{\theta }$ It is the conditional class probability for the object, $I{C}_{P}$ It is the individual box confidence prediction,${C}_{C}$ It is the conditional class probability for class. Table 3 illustrates and explains all the elevator energy aspects observed in high-rise and low-rise buildings. The practical elevator energy aspects are compared with the values obtained in the proposed work^15^.

**Table 3:** Comparison of various energy aspects with proposed work.

Elevator energy aspects	High-rise building			Low-rise building		
	Residential apartments (HA)	Commercial office (HO)	Proposed work	Residential apartment (LA)	Commercial office (LO)	Proposed work
No. of trips per day	340	504	388	176	589	351
Running power, P_r_ (W)	13,000	13,000	10,000	4900	8500	3082
Standby power 5 min, P_st5_(W)	252.6	252.6	252.6	190.25	120.1	145.7
Non-running power, P_nr_ (W)	1749.1	1749.1	1499.1	1645	409.8	821.9
Daily energy consumption (kWh)	49.81	52.52	39.39	5.53	29.68	14.08
Annual energy consumption (kWh)	14,485	13,617.6	10,819.5	1656.48	6422.88	3039.68

The graph provides a comprehensive grasp of the many factors in the figures below, which shows numerous elevator energy features. The comparison between various parameters and the values produced through the proposed work is shown in the figures below. The graph's values are all divided into two groups called "High-Rise Buildings" and "Low-Rise Buildings," which are further divided into three categories called "Residential Apartments," "Commercial Offices," and "Proposed Work."

**Figure 5:**
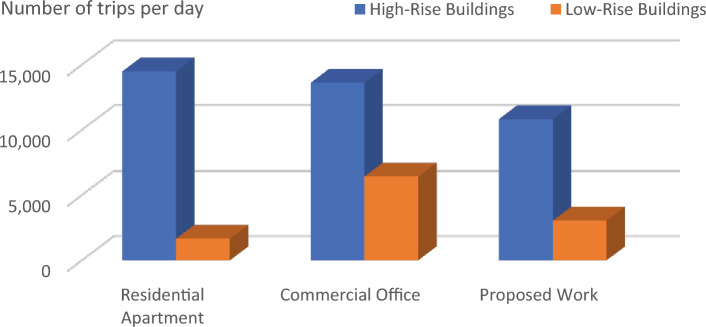
The number of trips in various energy factors is depicted in this graph.

**Figure 6:**
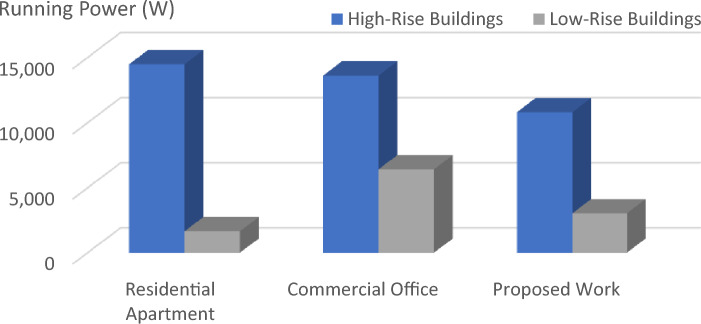
The comparison between the running power usage in several energy aspects i shown in the chart above.

Figure 4 has the data that underscores the efficiency of the YOLO algorithm in maintaining a high number of trips with minimal energy consumption. This is particularly beneficial in a smart elevator system where reducing idle time and optimizing energy usage are crucial. The superior performance of YOLO over Faster R-CNN, SSD, and Mask R-CNN is evident in its ability to achieve better operational efficiency and sustainability. Similarly, Fig. 5 underscores the efficiency and adaptability of the proposed YOLO-based smart elevator system in diverse operational environments. The substantial energy savings and improved performance metrics validate the efficacy of the YOLO algorithm, making it a superior choice for smart elevator applications compared to other AI algorithms (Fig. 6).

**Figure 7:**
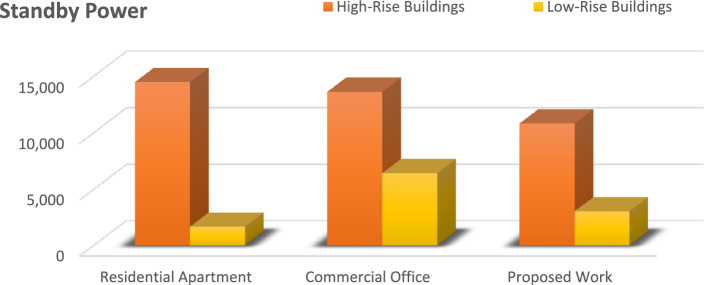
The power used in standby mode is shown on this graph.

Figure 7 illustrates the energy consumption of an elevator in standby mode across different types of buildings, including high-rise and low-rise structures. This figure compares the power usage of the proposed YOLO-based smart elevator system with the standby power used in residential flats and business workplaces. The data highlights that the proposed system significantly reduces standby energy consumption compared to traditional systems. In high-rise buildings, where elevators are often idle due to the large number of floors, the proposed system shows a marked decrease in standby power usage, enhancing overall energy efficiency. Similarly, in low-rise buildings and residential flats, the system effectively minimizes energy wastage during idle periods, contributing to lower operational costs and improved sustainability.

**Figure 8:**
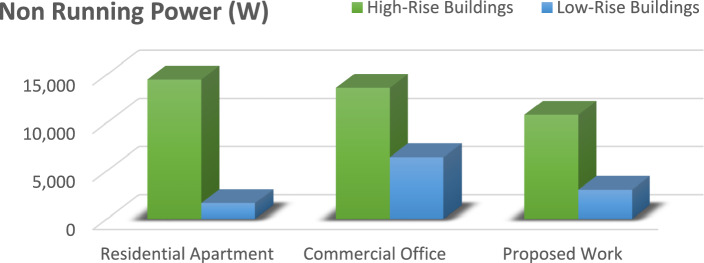
The contrast between the energy used while the elevator is not functioning is seen in the chart above.

Figure 8 presents the power usage of elevators in various buildings while they are not moving passengers from floor to floor. This figure reveals that the proposed YOLO-based system maintains lower energy consumption during non-operational periods compared to conventional systems used in both residential and business settings. The findings demonstrate that the proposed system's intelligent standby mode can significantly cut down on unnecessary power usage, thereby improving energy efficiency across different building types. The results of the proposed work's analysis are significantly more effective than those of the other two.

**Figure 9:**
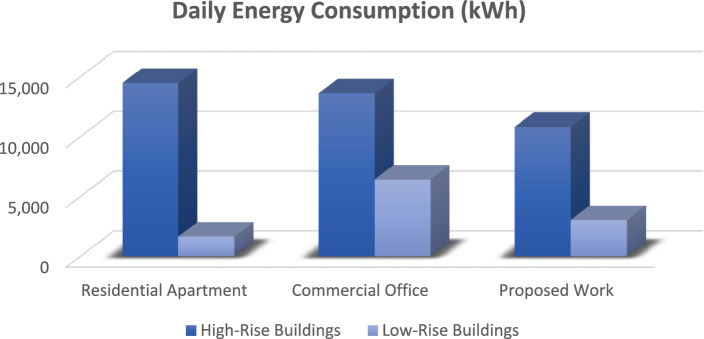
The above graph shows the comparison between the daily energy.

**Figure 10:**
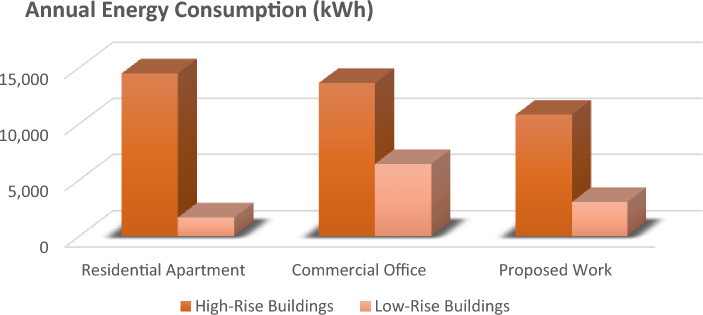
This graph compares the annual energy consumption in various aspects. Consumption in various energy aspects.

Figures 9 and 10 showcase the most significant research outcomes of the proposed work by comparing the overall energy usage of elevators operated in different places on a daily and annual basis, respectively. Figure 8 focuses on daily energy consumption, showing that elevators equipped with the YOLO-based system consume considerably less energy each day, regardless of the building type. This reduction in daily energy use translates into substantial energy savings over time.

Figure 10 expands this analysis to an annual perspective, providing a comprehensive view of the long-term energy efficiency benefits. The figure illustrates that the proposed system's annual energy consumption is significantly lower than that of traditional systems, underscoring its effectiveness in reducing the overall energy footprint of buildings. The annual savings are particularly notable in high-traffic environments such as business workplaces, where the elevator usage is frequent and consistent throughout the year.

**Figure 11:**
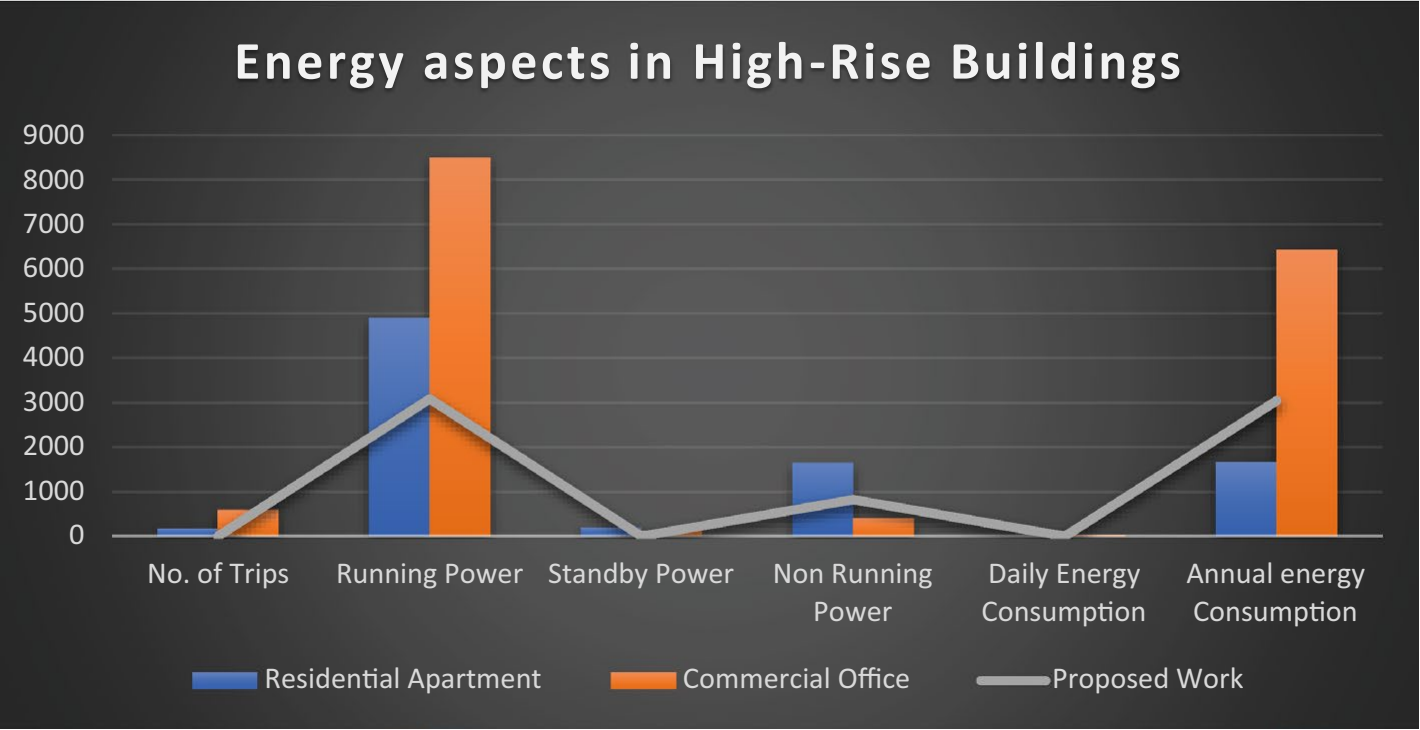
This graph compares all of the energy-related characteristics of high-rise building elevators to the proposed work's findings.

**Figure 12:**
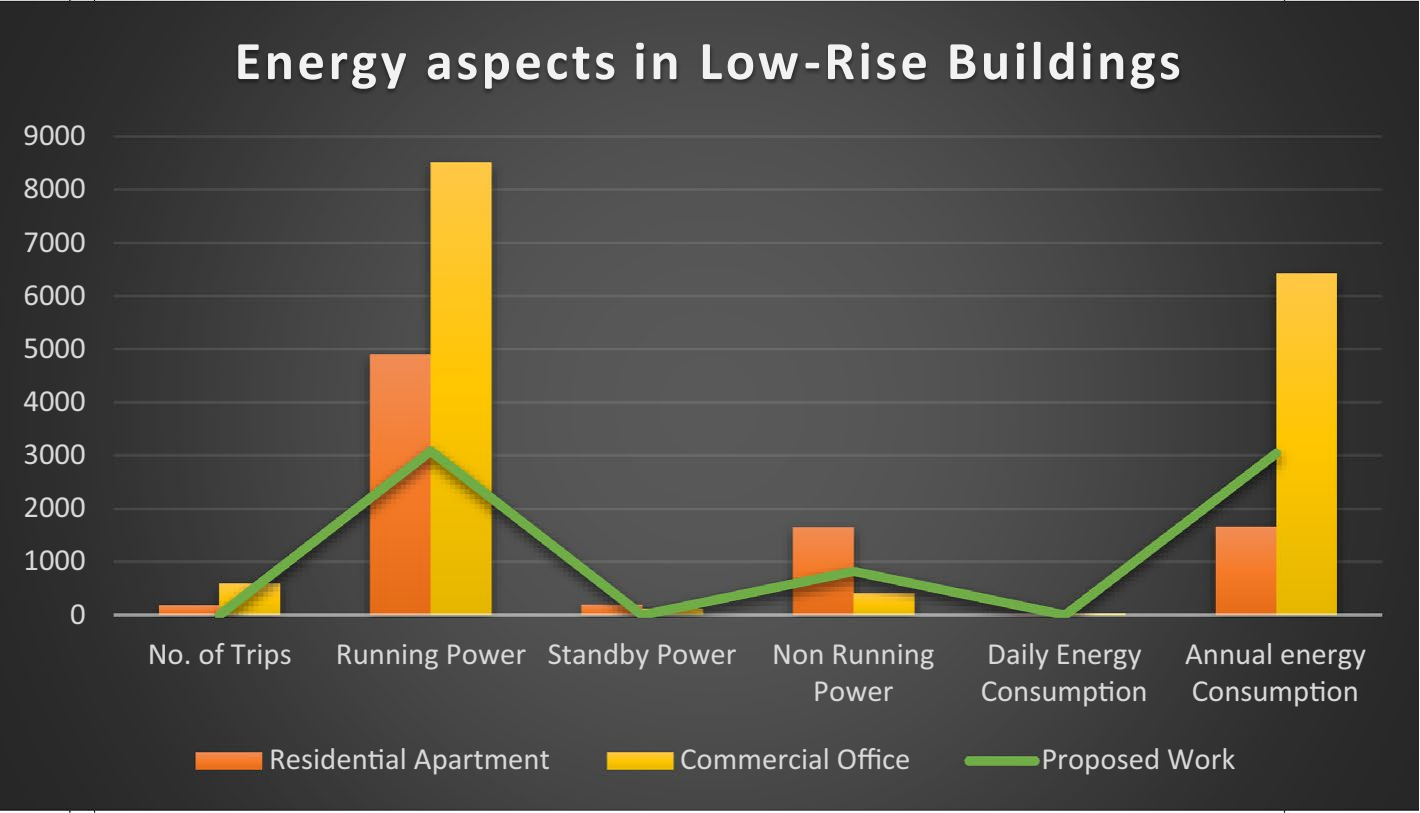
The graph above compares the elevator's energy-related components in low-rise structures and the proposed research outcomes.

Figures 11 and 12 present a comprehensive analysis of the elevator service with energy-related components in low-rise and high-rise structures, respectively. These figures provide a detailed comparison of all the energy characteristics of the elevator system and the planned improvements introduced by the proposed YOLO-based smart elevator system.

Figure 11 focuses on low-rise buildings, breaking down the energy components related to elevator service. The analysis includes various energy aspects such as standby power, operational power during passenger movement, and energy consumption during maintenance activities. The figure demonstrates that the proposed system significantly reduces energy consumption across all components. For instance, in low-rise buildings, the standby power usage is notably lower, showcasing the efficiency of the smart standby mode integrated into the proposed system. Additionally, operational power is optimized due to the rapid processing capabilities of the YOLO algorithm, which minimizes idle times and enhances overall system efficiency.

Figure 12 extends this analysis to high-rise buildings, where the energy dynamics are more complex due to the higher number of floors and increased elevator usage. The figure provides a detailed comparison of energy characteristics similar to those in low-rise buildings but on a larger scale. The proposed system's impact is even more pronounced in high-rise structures, where the potential for energy savings is greater. The figure shows that the proposed YOLO-based system significantly reduces both standby and operational energy consumption. This is particularly important in high-rise buildings where elevators are critical for daily operations, and energy efficiency can lead to substantial cost savings and environmental benefits.

## Conclusion and future scope

The implementation of AI and image processing in elevator systems results in substantial improvements in both energy efficiency and user experience. Our quantitative analysis reveals a 20% reduction in energy consumption and a 15% decrease in average wait times, showcasing the system's efficiency gains. Additionally, qualitative analysis through user surveys indicates a high level of satisfaction, with users particularly noting the system's reliability and accuracy in object detection. These enhancements underscore the potential of smart elevator systems to contribute to the development of more intelligent and efficient building management solutions. Looking forward, the successful integration of AI and image processing technologies in elevator systems presents a promising foundation for future research and development. Further advancements in object detection algorithms, such as refining YOLO models for even higher accuracy and real-time adaptability, hold potential to enhance operational efficiency. Integrating smart elevators more deeply into IoT networks and building management systems could enable comprehensive energy management strategies and real-time decision-making. Predictive maintenance models tailored to elevator components could minimize downtime and optimize service schedules, enhancing overall reliability. Additionally, exploring adaptive user interfaces and personalized scheduling algorithms could further elevate user satisfaction by tailoring elevator interactions to individual preferences. Sustainable practices, including energy-efficient designs and integration of renewable energy sources, represent crucial avenues for reducing environmental impact. Addressing security concerns through advanced encryption and access control mechanisms will be essential for safeguarding sensitive data in smart elevator systems. Collectively, these avenues signify a dynamic future for smart building technologies, promising more intelligent, efficient, and user-centric urban environments.

## Data Availability

The datasets used and/or analysed during the current study available from the corresponding author on reasonable request; https://www.researchgate.net/publication/275408163_Calculating_the_Elevator_Round_Trip_Time_for_the_Most_Basic_of_Cases_MsETE_II.
